# Miscellany

**Published:** 1891-12

**Authors:** 


### Miscellany.

THE *Obstetrical Record*, published by the New York Pharmacal Association, comprises a chart for the accurate report of obstetric cases. It aims to secure (1) a complete history of the patient, her age, name, nativity, residence, occupation, religion, married or single. 2. Previous labors, their duration, operative interference, complications, sequelæ, lactation and its complications, condition of child. 3. Present pregnancy, with time of quickening; expected date of confinement; examination of urine; examination, vaginal and by palpation, pelvimetry, complications and general therapeutic measures. 4. Present labor, date, duration, anesthetic used and character of pains, presentation and position, operative measures and complications. 5. A chart for the temperature and pulse, after-treatment, lactation, sex, condition of child and diameter of fetal head.

The object of this record is to encourage the accoucheur in the habit of accurate note-taking. We commend the design, and think the plan here presented will, by its simplicity, prove a ready help to the busy practitioner in making a complete history of his obstetric cases. If such a chart could be more generally adopted and carefully filled out, we would have more scientific work and better and more reliable statistics in obstetrics.

---

All Around the Year 1892.—Entirely new design, in colors, by J. Pauline Sunter. Printed on heavy cardboard, gilt edges, with chain, tassels, and rings. Size, 4½ by 5½ inches. Boxed. Price, 50 cents. Lea & Shepard, Boston, Mass.

This is a most charming calendar, tastily tied with a white silk cord, with a delicate silver chain attached. The cards representing the months are arranged on rings, so that they can be turned over as each month is needed for reference. It is as fresh in design as it is fresh in the fair whiteness of its workmanship. Each card contains not only the calendar, but a design both charming and appropriate, and an equally timely sentiment. The drawings are in Mrs. Sunter's picturesque style, and executed in sepia tint and color.

Altogether it is a charming piece of work, a thorough pleasure to the eye, and sure to win a welcome wherever it goes.

